# Regulation of Synthetic Biology: Developments Under the Convention on Biological Diversity and Its Protocols

**DOI:** 10.3389/fbioe.2020.00310

**Published:** 2020-04-09

**Authors:** Felicity Keiper, Ana Atanassova

**Affiliations:** ^1^BASF Australia Ltd., Southbank, VIC, Australia; ^2^BASF Belgium Coordination Center, Technologiepark-Zwijnaarde, Ghent, Belgium

**Keywords:** synthetic biology, living modified organisms, Cartagena Protocol on Biosafety, Nagoya Protocol on Access and Benefit Sharing, risk assessment, gene drives, digital sequence information, biotechnology regulation

## Abstract

The primary international forum deliberating the regulation of “synthetic biology” is the Convention on Biological Diversity (CBD), along with its subsidiary agreements concerned with the biosafety of living modified organisms (LMOs; Cartagena Protocol on Biosafety to the CBD), and access and benefit sharing in relation to genetic resources (Nagoya Protocol to the CBD). This discussion has been underway for almost 10 years under the CBD agenda items of “synthetic biology” and “new and emerging issues relating to the conservation and sustainable use of biological diversity,” and more recently within the scope of Cartagena Protocol topics including risk assessment and risk management, and “digital sequence information” jointly with the Nagoya Protocol. There is no internationally accepted definition of “synthetic biology,” with it used as an umbrella term in this forum to capture “new” biotechnologies and “new” applications of established biotechnologies, whether actual or conceptual. The CBD debates are characterized by polarized views on the adequacy of existing regulatory mechanisms for “new” types of LMOs, including the scope of the current regulatory frameworks, and procedures and tools for risk assessment and risk mitigation and/or management. This paper provides an overview of international developments in biotechnology regulation, including the application of the Cartagena Protocol and relevant policy developments, and reviews the development of the synthetic biology debate under the CBD and its Protocols, including the major issues expected in the lead up to and during the 2020 Biodiversity Conference.

## Introduction

The world of “synthetic biology” is an optimistic and ambitious one, with its claims of transformative and paradigm-shifting developments, and promises of providing solutions for global challenges such as food security, energy security, clean water, human and animal health, environmental contamination, species conservation, and even climate change (Ro et al., 2006; Khalil and Collins, 2010; Redford et al., 2013; Kelley et al., 2014; Organisation for Economic Cooperation and Development [OECD], 2014; Redford, 2014; Sliva et al., 2015; Crow, 2018; Gray et al., 2018). These promises appeal to research funders, fascinate the public and even inspire the next generation of scientists, however they also arouse fear in a society where biotechnology is often perceived as controversial. Whether or not “synthetic biology” could contribute toward these global challenges depends not only on the scientific realities matching the hype (Kwok, 2010; Cameron et al., 2014; Ostrov et al., 2019), but also the interconnected issues of the regulatory environment and societal acceptance.

Synthetic biology is part of the continuum of modern biotechnological development that commenced with the emergence of molecular cloning, recombinant DNA technologies and the polymerase chain reaction from the early 1970s and through the 1980s (Berg et al., 1974; Cameron et al., 2014). These technologies enabled the modification and intentional transfer of DNA from one organism into another and were perceived as truly paradigm-shifting. The developments in biotechnology in the 1970s were accompanied by both excitement and concerns about the potential risks. In response to the latter, in 1974 the scientific community recommended “voluntarily deferring” certain types of laboratory experiments until an international scientific discussion could be held to review scientific progress and examine the potential risks and how to manage them (Berg et al., 1974). In 1975 the Asilomar Conference on Recombinant DNA Molecules was attended by some 140 scientists, predominantly from public institutions from around the world, as well as lawyers, government officials and members of the media. At Asilomar it was agreed that the research should continue but with appropriate safeguards in place, thus heralding the beginning of precautionary biosafety regulation in this field (Berg et al., 1975; Berg and Singer, 1995; Berg, 2008).

The concerns about the risks of recombinant DNA and associated “new” technologies evident in the 1970s persist in present-day regulatory policy debates, and with the beginning of the current millennium there were calls for another “Asilomar” for “synthetic biology” (Brenner and Sismour, 2005). For those who witnessed developments in the 1970s, the current debates are a case of history repeating itself, with the same range of views expressed: from biotechnological developments being inherently risky and requiring stringent regulation based on the precautionary approach, through to these technologies not presenting unique or novel risks. The latter view is held predominantly by members of the scientific community who point out that much of “synthetic biology” is congruent with the technologies discussed in Asilomar in the 1970s, and that a substantial body of scientific evidence has accumulated over the past four decades with no documented hazard to public health attributed to products of these technologies (Berg and Singer, 1995; Brenner and Sismour, 2005; National Academies of Sciences Engineering and Medicine [NASEM], 2016a). However, in today’s debate it is evident that concerns about the adequacy of regulation conflate broader political and societal issues beyond the safety of the technologies and their products.

**FIGURE 1:**
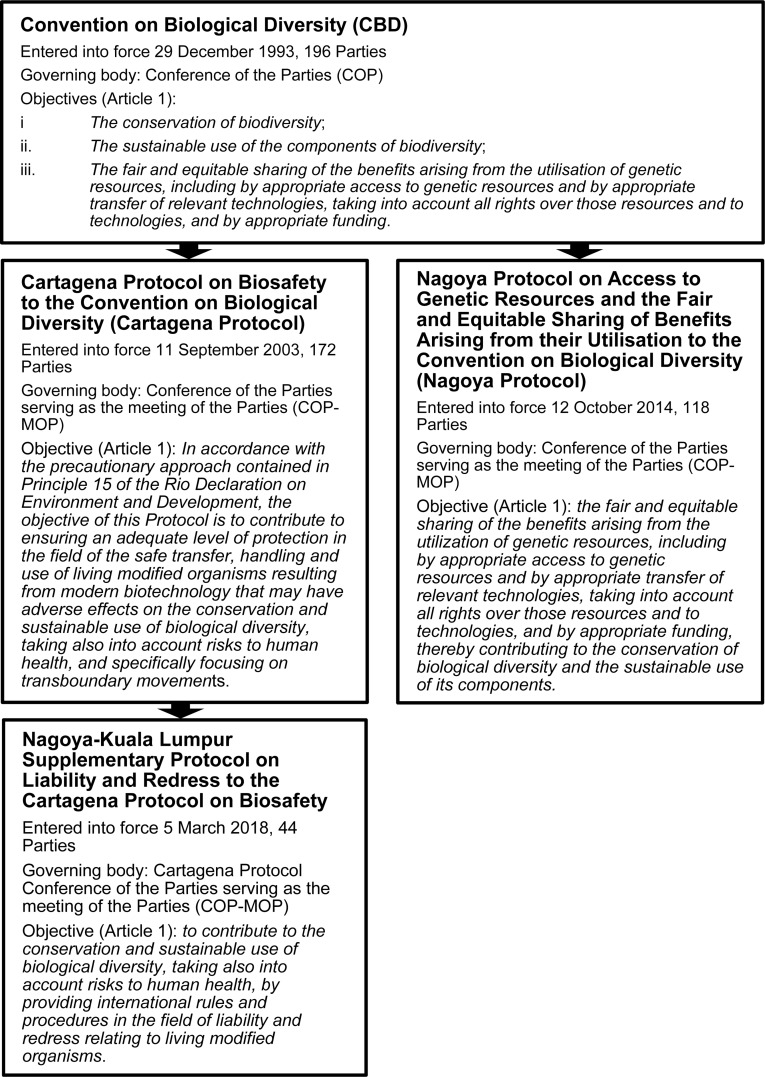
The objectives of the CBD and its subsidiary treaties.

**FIGURE 2:**
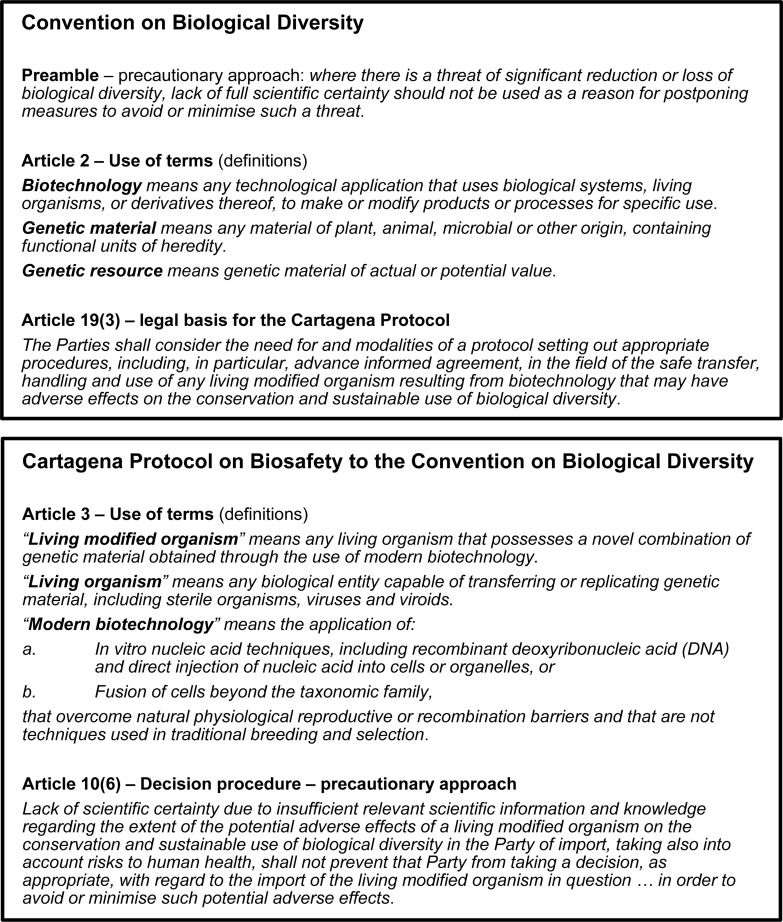
Key provisions of the CBD and the Cartagena Protocol.

At the international level, the United Nations Convention on Biological Diversity (CBD)^1^ was one of several significant environment-related outcomes of the 1992 Earth Summit in Rio De Janeiro^2^. The CBD is ratified by 196 countries (“Parties”), which include all countries of the world except for the United States of America (USA) and the Holy See^3^. The objectives of the CBD (and its subsidiary treaties) are set out in Figure 1. During the drafting of the CBD, the potential for biotechnology to contribute to its objectives was recognized, provided that adequate safety measures were applied to its development and use (Secretariat of the Convention on Biological Diversity [SCBD], 2003). The resulting treaty obligates Parties to “regulate, manage or control the risks associated with the use and release of living modified organisms resulting from biotechnology which *are likely to have* adverse environmental impacts…” (emphasis added) [Article 8(g)]. It also provides the legal basis for a supplementary protocol [Article 19(3); see Figure 2] which CBD Parties started negotiating in 1995 (COP2; Decision I/9) and adopted in 2000 as the CBD’s first subsidiary agreement, the Cartagena Protocol on Biosafety to the CBD (“Cartagena Protocol”) (Secretariat of the Convention on Biological Diversity [SCBD], 2000). The Cartagena Protocol sets out a regulatory framework for the safe use, handling and transfer of living modified organisms (LMOs; analogous to genetically modified organisms/GMOs) (Secretariat of the Convention on Biological Diversity [SCBD], 2003). Some key provisions and definitions of the Cartagena Protocol that impact on the CBD synthetic biology debate are set out in Figure 2.

In addition to the Cartagena Protocol, the CBD has produced a second subsidiary agreement, the Nagoya Protocol on Access and Benefit Sharing to the CBD (“Nagoya Protocol”) (Secretariat of the Convention on Biological Diversity [SCBD], 2011a), with the Cartagena Protocol also having a supplementary protocol on the topic of liability and redress (see Figure 1; Secretariat of the Convention on Biological Diversity [SCBD], 2011b). These two treaties are not the focus of this review, however they are relevant to the overall international biotech regulatory framework. The CBD and each of the Protocols have their own governing bodies, and since 2016 these have met in concurrent sessions during a 2-week “Biodiversity Conference.”

At the time of writing (February 2020), several programs of work are in progress on various CBD and Protocol issues with relevance to synthetic biology, the outcomes of which will be considered by major meetings of CBD subsidiary bodies in May 2020 and the treaty governing bodies in October 2020 at the biannual Biodiversity Conference. Some of these issues are also under consideration as part of an extensive preparatory process underway for the development of the “Post-2020 Global Biodiversity Framework” that is expected to be adopted at the Biodiversity Conference in October 2020. While synthetic biology is a CBD issue, it has overlap with other issues under the CBD’s subsidiary protocols, as well as aspects of the Post-2020 Global Biodiversity Framework. This paper provides an overview of major developments in biotechnology regulation and relevant policy developments, examines what “synthetic biology” is, and reviews the development of the synthetic biology debate under the CBD and its Protocols, including the major issues expected in the lead up to and during the Biodiversity Conference in 2020. To begin, a brief overview is provided of the CBD treaty processes that form the basis of this discussion.

## CBD Processes – a Primer

To date, the CBD has generated 435 decisions at fourteen meetings of its governing body^4^, the COP, and the fifteenth meeting of the COP (COP15) will be held in China in October 2020. The work of the COP is supported by two CBD subsidiary bodies: the Subsidiary Body on Scientific, Technical, and Technological Advice (SBSTTA)^5^ and the Subsidiary Body on Implementation (SBI)^6^. The SBSTTA has met 23 times to date, with the twenty-fourth meeting (SBSTTA24) scheduled for May 2020, and synthetic biology is on the provisional agenda of that meeting^7^.

Meetings of the SBSTTA may be described as “mini-COPs” because the outcomes from an increasing number of programs of work referred to it by the COP are deliberated and SBSTTA produces recommendations that become the basis (draft decisions) for the negotiations and subsequent decisions of the COP. For synthetic biology, SBSTTA24 is expected to address the COP14 request to *consider* the outcomes of a program of work that consists of submissions of information on a series of synthetic biology topics^8^, a series of online discussions on those topics held in March 2019^9^, and a report from the *Ad Hoc* Technical Expert Group (AHTEG) on Synthetic Biology that met in June 2019^10^. The SBSTTA24 is also requested to contribute to the completion of the analysis required to indicate whether or not synthetic biology qualifies as a “new and emerging issue” (NEI; Decision XIV/19). The latter refers to one of the functions of the SBSTTA to identify “new and emerging issues relating to the conservation and sustainable use of biodiversity,” and this is discussed in further detail in section Synthetic Biology Under the CBD below.

The two Protocol COP-MOPs are also able to refer work to the CBD’s SBSTTA, and for SBSTTA24 this includes the Cartagena Protocol topic of LMO risk assessment and risk management (Articles 15 and 16, Annex III), which has overlapping scope with synthetic biology^11^. An issue that arose from synthetic biology discussions that is now under consideration jointly by the CBD and the Nagoya Protocol is “digital sequence information” (DSI). This topic is under discussion within the Post-2020 Global Biodiversity Framework development process, which will also be on the agenda of SBSTTA24. All of these topics are discussed further in section Synthetic Biology Under the CBD below.

The COP and COP-MOP decisions, recommendations of the SBSTTA, reports of *Ad Hoc* Technical Expert Groups (AHTEGs), submissions of information, online discussions and NEI proposals that are referred to in this review are all available and accessible online via the CBD website^12^. Document, decision and recommendation numbers and weblinks are provided throughout.

## The Cartagena Protocol

The Cartagena Protocol applies to any living organism that possesses a novel combination of genetic material *obtained using modern biotechnology* (emphasis added) (Article 3; see Figure 2). In other words, regulation of the organism is triggered by the use of modern biotechnology, which amounts to process-based regulation (Atanassova and Keiper, 2018). At the time the Cartagena Protocol and national and regional frameworks were drafted, process-based triggers may have provided a clear distinction between organisms within or outside of the scope of regulatory oversight. However, as biotechnology has evolved over time, distinctions have blurred and the continuing suitability of definitions developed in the 1980s and 1990s are questioned. For example, if the Cartagena Protocol’s definition of “modern biotechnology” was strictly applied to take into account the need for overcoming “natural physiological or reproductive or recombination barriers and that are not techniques used in traditional breeding and selection,” some recombinant DNA (e.g., cisgenesis) and “new” technologies (e.g., genome editing) may be excluded from its scope. Such definitions have given rise to debate in countries throughout the world on the regulatory status of “new techniques” such as genome editing, and this is one of the issues underlying the CBD discussions on synthetic biology (Atanassova and Keiper, 2018). In practice, Parties to the Cartagena Protocol differ in their interpretation and implementation of its definitions, with regulatory systems ranging from being largely process-based (e.g., European Union) to mostly product-based (e.g., Japan) (Nap et al., 2003).

Differences in implementation of the Cartagena Protocol also arise due to the primacy given to the precautionary approach which is introduced in the preamble of the CBD, and the Cartagena Protocol provides for its application in regard to making decisions about the import of LMOs [Article 10(6); see Figure 2]. It is a controversial feature of the Cartagena Protocol because it can lead to unpredictability in how biotech regulation is implemented; in highly risk-averse societies, the precautionary approach may be invoked to refuse the introduction of an LMO into the environment in the absence of identified risks if there is any doubt about its potential effects (Conner et al., 2003). A process-based trigger for LMO regulation is consistent with a precautionary approach, as its basis lies in the presumption that the technology is inherently risky, with all organisms resulting from biotech captured within regulatory scope regardless of their characteristics and the actual risks (if any) they present.

Another key feature of the Cartagena Protocol is the “advance informed agreement” procedure, which requires countries to be provided with the information necessary to enable them to undertake a risk assessment before deciding whether or not to permit the import of an LMO for intentional release into the environment (Articles 7, 10, 15, and Annex III). The principles of risk assessment set out in the Cartagena Protocol are found in biotech regulatory frameworks around the world, irrespective of whether or not the country is a Party to the Cartagena Protocol, as these principles were influenced by prior international guidance on the topic (e.g., Organisation for Economic Cooperation and Development [OECD], 1986, 1992, 1993) as well as the experience of countries already assessing LMOs for environmental release. Further, in practice any decision to release an LMO into the environment is informed by risk assessment, even when there is no transboundary movement associated with the release.

It should be mentioned that several countries who are major agricultural producers and exporters are not Parties to the Cartagena Protocol. Many countries developed biotech regulatory systems (e.g., Australia, Argentina, Brazil, China, the European Union, India, and South Africa) or adapted their existing legislative regimes (e.g., Canada and the USA) for the purpose of identifying and managing risks posed by GMOs to human health and the environment in parallel to the drafting of the CBD in preparation for the first releases of GM crops into the environment from the late 1980s and through the 1990s (Nap et al., 2003). Some of those countries became Parties to the Cartagena Protocol, but those that have not are still Parties to the CBD (except for the USA) and able to fully participate in discussions and decision-making and contribute their relevant expertise in the ongoing CBD synthetic biology discussions. These countries can participate in discussions under the Cartagena Protocol as “other governments” however they are not able to directly participate in decision-making under that treaty.

## What Is “Synthetic Biology”

Synthetic biology is often reported to have emerged as a field of biotechnology in the early 2000s, with the convergence of engineering principles with biology (European Academies Science Advisory Council [EASAC], 2010). This time saw the first international meetings on “synthetic biology” and the beginning of the International Genetically Engineered Machine (iGEM) competition (Gray et al., 2018). It also saw the emergence of a new lexicon that reflected engineering concepts, e.g., standardized “parts” (such as “Biobricks”), chassis, circuits, plug-and-play; and claims of a new scientific culture of greater collaboration facilitated by the standardization of processes across labs, and openness and ethical awareness (Crow, 2018). The rapid adoption of the term “synthetic biology” may have been facilitated by its use by a self-defining new generation of researchers from a variety of disciplines (Oldham et al., 2012) seeking to differentiate themselves from past controversies, and also avoid issues with attracting funding and public acceptance. However, the high-profile publication of the first self-replicating bacterial cell with a fully synthetic “computer-designed” chromosome in 2010 (Gibson et al., 2010), which was accompanied by headlines proclaiming that man had created life^13^, returned the spotlight on biotechnology regulation, and triggered new discussions on international regulatory oversight under the CBD.

**FIGURE 3:**
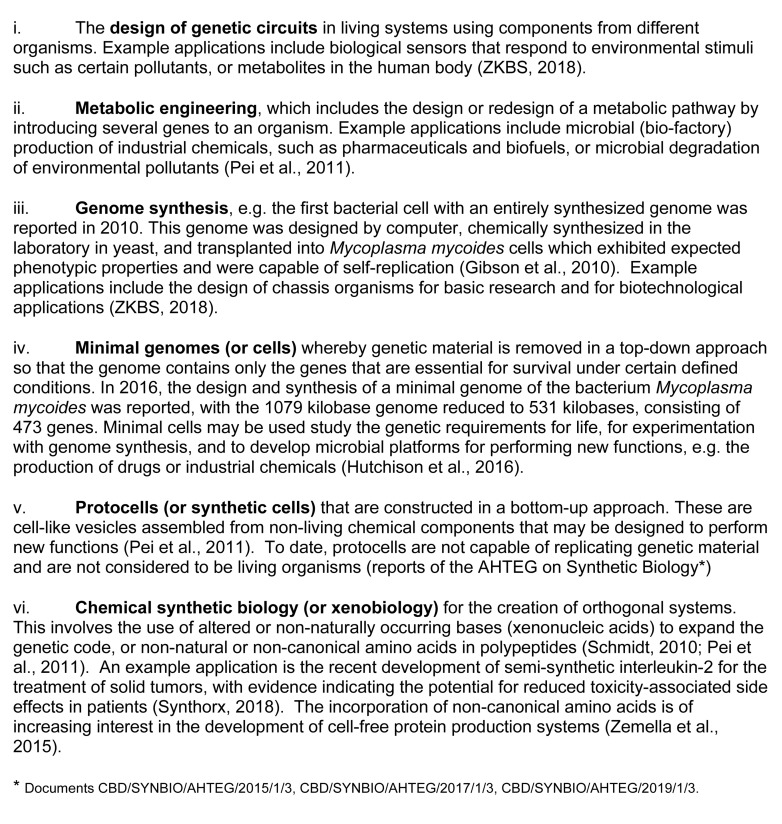
Sub-fields of biotechnology that may be referred to as synthetic biology in the scientific literature.

Today many definitions and descriptions of synthetic biology can be found, but there are none that are universally agreed or applicable to everything that may be labeled as such in the CBD discussions. Fields or sub-areas of biotechnology that may be referred to as synthetic biology in the scientific literature and example applications are listed in Figure 3. At its simplest, synthetic biology may be described as combining DNA or genetic “parts” in novel configurations to modify existing properties or to create new ones (Oldham et al., 2012). More broadly, there is a general consensus that synthetic biology is a dynamic and growing area of biotechnology that utilizes accumulated and constantly advancing knowledge and understanding in biological engineering, and advancements in engineering tools (Raimbault et al., 2016). As for recombinant DNA technologies (Berg and Singer, 1995), synthetic biology is expected to have a profound impact on our knowledge of fundamental life processes. It is also expected to improve on and expand the range of potential biotechnological applications. Since the 1980s, recombinant DNA technologies have delivered products including drugs, industrial products and improved agricultural varieties (Berg and Singer, 1995). The focus of synthetic biology applications thus far include the production of pharmaceuticals and vaccines, and to provide alternatives to fossil-based fuels and relieve pressure on non-renewable resources (German Central Committee on Biological Safety [ZKBS], 2018; United Nations Environment Programme [UNEP], 2019).

While the use of the term synthetic biology is clearly broad and likely to evolve further with the advancement of technical and scientific knowledge, a challenge exists for regulators to distinguish what is truly new and not within the scope of existing applicable regulatory mechanisms. Identifying regulatory “gaps” is important as it allows regulations to be adapted to scientific progress. Distinctions that emerged early in the synthetic biology dialogue were based on expectations of unprecedented engineering complexity or scale, and speed (see the 2015 report of the AHTEG on Synthetic Biology^14^; European Academies Science Advisory Council [EASAC], 2010; Kuzma and Tanji, 2010). However, almost 10 years of work under the CBD (see section Synthetic Biology Under the CBD below) has failed to identify a LMO that would not be within the scope of the Cartagena Protocol, regardless of the complexity of the actual (or conceptual) genetic modification. In connection to the speed of development of the technology and the resulting organisms, countries have raised concerns about having the necessary resources to adequately assess and manage anticipated risks, however, regulators participating in the CBD discussions have not indicated that they have been inundated with such applications. More broadly, new types of LMOs may present resource and capacity challenges for less experienced regulators, and developing country Parties who have not yet, or are still in the process of, implementing the Cartagena Protocol at the national level.

Another often proposed distinction between recombinant DNA technology and synthetic biology is that the former involves the transfer of individual genes, whereas the latter involves the assembly of new DNA sequences (Science Communication Unit UWE, 2016). While this division is more technically specific, it remains an overly simplistic and inaccurate representation of recombinant DNA technologies as merely for “cut and paste,” and of synthetic biology as a tool for generation of new DNA sequences. Both are based on common enabling technologies and involve the assembly of DNA sequences that are based on/are analogous to existing genetic material, and involve the transfer of genetic material into an existing living recipient cell/host. Thus, different views persist as to whether certain synthetic biology applications (particularly genetic circuits, metabolic engineering and genome synthesis; listed in Figure 3) are fundamentally new or are merely advances along the biotechnology continuum. There are examples of transgenic crops that are tagged by some with the synthetic biology label, particularly those that are examples of “metabolic engineering,” e.g., Golden Rice that produces pro vitamin A, and crops that produce higher levels of omega-3 fatty acids. However, the promise of nutritionally enhanced crops, and work on developing Golden Rice, began with the dawn of plant genetic engineering in the early 1980s. The development of omega-3 crops followed in the 1990s (Enserink, 2008; Napier et al., 2019), with the first regulatory approvals in support of commercial cultivation of oilseed rape obtained in 2018^15^. Similarly, the design and construction of gene constructs using well characterized elements (referred to as “parts” by practitioners of synthetic biology), which are used in long-commercialized GM crops, are consistent with synthetic biology “approaches.” Such innovative products may appear novel when they are ready for market, however their development may have taken 30 years (Napier et al., 2019).

### Genome Editing

An area of technological development that is often linked with synthetic biology is the broad category of enabling tools for genome editing, in particular the technology known as “CRISPR” (clustered regularly interspaced short palindromic repeats) (Jiang and Doudna, 2017), and existing or conceptual applications of CRISPR such as organisms containing engineered gene drives (Legros et al., 2013), *de novo* domestication of species (Zsögön et al., 2018), and multiplex editing (Scientific Committees, 2014; Sánchez-León et al., 2017; Borrelli et al., 2018; Feng et al., 2018; Guo et al., 2018; Li A. et al., 2018; Li L. et al., 2018; Zsögön et al., 2018). Describing genome editing as synthetic biology is difficult to reconcile with the many descriptors of synthetic biology, particularly when considering the various potential outcomes of genome editing. For example, in crop breeding the outcomes of genome editing range from DNA sequence changes that are comparable to the outcomes of spontaneous or induced mutations, to targeted gene insertions which are comparable to transgenic crops (Custers et al., 2019). Therefore, in essence the outcomes of genome editing in crops are comparable to existing biotech and non-biotech approaches for generating genetic variation. Of note, in their assessment, three Scientific Committees advising the European Commission reported that multiplexed genome editing allows for genome-wide modification in a way that is more accurate and precise than changes made using conventional methods. They considered that it is the ease of using the technology and potential speed of development of new organisms that could present regulatory challenges in terms of adequate risk assessment (Scientific Committees, 2015b), rather than the technology or characteristics of the resulting organisms.

## Policy Discussion on Synthetic Biology Regulation

The initial developments in “synthetic biology” were mostly centered in the USA and in Europe, led by the United Kingdom, Germany, Switzerland and France. The USA remains the world leader in terms of research entities and investment in research and development (Pei et al., 2011). Expansion elsewhere in the world has been driven by the opportunities for investment in research and development, as well as for socio-economic development. This is evident for example in several countries in Asia that have invested in the establishment of national synthetic biology initiatives that are contributing to advancements in the field, e.g., China, Japan, Korea, and Singapore (Chang, 2016; Ong, 2018).

The earliest synthetic biology policy discussions occurred in the USA, with similar timing to the beginning of synthetic biology discussions under the CBD, and these remain the most detailed investigations on technological developments under the umbrella of synthetic biology, their potential impacts and associated regulatory considerations. These are reviewed in brief below.

### National Policy Developments

#### United States of America (USA)

With the 2010 publication of the first self-replicating bacterial cell carrying an artificially synthesized and assembled genome (synthetic genome), then United States President Obama requested that the Presidential Commission for the Study of Bioethical Issues examine the implications of this emerging science. The resulting report concluded that the science posed limited risks, and there was no justification for a moratorium, or the development of new federal regulations, i.e., the existing regulatory mechanisms applicable to “genetically engineered organisms” remained relevant. The report made the important observation that the work did not amount to “creating life,” with this remaining a remote possibility for the foreseeable future; importantly, in this work the chemically generated genome was inserted into an already living naturally existing host cell (Presidential Commission for the Study of Bioethical Issues, 2010).

A series of reports followed from the National Academies of Sciences Engineering and Medicine (NASEM) addressing applications, products and enabling technologies that are included in the scope of “synthetic biology” CBD discussions. In their 2017 report on the “future products of biotechnology,” the NASEM reached the conclusion that the “…scale, scope, complexity, and tempo of biotechnology products are likely to increase in the next 5–10 years. Many products will be similar to existing biotechnology products, but they may be created through new processes, and some products may be wholly unlike products that exist today.” Such “similar” products include “next generation” GM crops, for which it was not anticipated that risk-assessment endpoints would be different from previously assessed GM crops. Less familiar products include gene drives designed to “suppress or enhance a species population at a rate that is faster than natural ecological processes or evolutionary rates”; such new products may require the definition of additional pathways to risk-assessment endpoints (National Academies of Sciences Engineering and Medicine [NASEM], 2017). These conclusions were broadly supported by NASEM reports published in the previous year that presented detailed reviews on the status of GM crops (National Academies of Sciences Engineering and Medicine [NASEM], 2016a) and gene drive research (National Academies of Sciences Engineering and Medicine [NASEM], 2016b). The NASEM emphasized the need for regulatory systems to have the agility to rapidly adapt to technological change and manage the assessment of a greater diversity of products (National Academies of Sciences Engineering and Medicine [NASEM], 2017).

The NASEM also examined the realistic capabilities of synthetic biology in the context of dual use. This is an issue long-connected to biotechnology that recognizes that while genetic engineering is predominantly pursued for beneficial purposes there is the possibility of it being applied for malicious use such as biological or chemical weapons. Their 2018 report on “biodefense in the age of synthetic biology” concluded that synthetic biology “expands the landscape of potential concerns,” e.g., by modifying the properties of existing microorganisms, using microorganisms to produce chemicals, or employing novel or unexpected strategies to cause harm. The report recommended that the USA should closely follow advances in the field and develop expanded strategies to prevent and respond to emerging biologically-enabled threats (National Academies of Sciences Engineering and Medicine [NASEM], 2018). Of note, one such strategy under investigation is the “Insect Allies” project funded by a research agency of the US Department of Defense^16^ which aims to use insects as a delivery tool for genetically modified viruses in order to address threats to food security by agricultural pests. This project has itself sparked dual use concerns amongst some researchers (Reeves et al., 2018), and concerns about new methods for “*in situ*” genetic modification in the CBD synthetic biology discussions.

#### United Kingdom (UK)

In the UK, a strategic roadmap (Roadmap) for synthetic biology was published in July 2012 (Technology Strategy Board, 2012) with the key purpose of developing “a roadmap that defines the likely timeframe and actions required to establish a world leading Synthetic Biology industry within the UK” (Clarke et al., 2012). The Roadmap was produced by an independent panel of experts for the government’s Department for Business Innovation and Skills, and it sets out a vision for realizing the potential of synthetic biology with a focus on economic success, the use of cutting-edge science, and clear public benefit. While the Roadmap is primarily focused on recommendations for funding and policy activities to support research and innovation, it considers the applicable regulation and governance systems, and emphasizes the need for responsible research and innovation within an effective, appropriate and responsive risk-based regulatory framework. Notably, the Roadmap points out that synthetic biology “operates within the existing regulatory framework” for GMOs at the international (Cartagena Protocol), regional (applicable European Directives) and national (UK) levels, and the general consensus amongst regulators that these remain broadly adequate but a “watching brief” should be maintained as technology continues to develop.

In 2015, the UK Synthetic Biology Strategic Plan 2016 (Synthetic Biology Leadership Council, 2015) was released that built upon the 2012 Roadmap. It provided stronger focus on the responsible acceleration of commercial delivery of new products and services of public benefit and emphasized again the need for responsible research and innovation, and proportionate and adaptive regulation for the maximization of public benefit and minimization of risk. It also suggests the development of technical standards at the national level to support the acceleration of commercialization (The British Standards Institution, 2015). These standards could also assist regulators, and support the UK in contributing to international discussions on appropriate regulatory and governance systems for synthetic biology.

Both the 2012 Roadmap and 2016 Strategic Plan briefly consider the issue of dual use, with the latter pointing out that guidelines and regulatory processes exist for accidental or deliberate misuse, that these are broader than synthetic biology, and that they need to be kept under review. The Strategic Plan also considers that synthetic biology tools have a key role in defending the UK against such incidents and regulatory systems need to enable rapid response.

#### Germany

Reports from other countries have been published more recently, with that by the German Central Committee on Biological Safety (ZKBS) in 2018 concluding that most synthetic biology approaches result in GMOs that can be assessed according to the existing German regulatory framework, the applicable European Directives (2001/18/EC and 2009/41/EC), and the Cartagena Protocol. Specifically, their assessment concluded that the insertion of synthetic genes, gene circuits, metabolic pathways, or entire genomes in an organism results in a GMO as defined by these regulatory frameworks. They also concluded that the reduction of a genome to create a minimal cell, and the use of xenonucleic acids to create bio-orthogonal systems are approaches that result in GMOs within the scope of existing regulatory frameworks. Further, they concluded that these developments did not present specific risks in addition to those already assessed for GMOs developed using recombinant DNA technologies (German Central Committee on Biological Safety [ZKBS], 2018).

#### Australia

Similarly, in October 2018 the final report produced following a review of the national regulatory framework by the Federal Government in Australia concluded that synthetic biology remains within its scope. In that review, synthetic biology was described as a “broad range of techniques, applications and products” that are “not qualitatively different” from that already regulated by the framework, but it was recommended that a “watching brief” be maintained to ensure this remained the case with future developments (Department of Health, 2018). This conclusion is consistent with the earlier advice of the Gene Technology Ethics and Community Consultative Committee – the committee that provides advice to the Office of the Gene Technology Regulator – in 2013 that synthetic biology did not raise new technical (or ethical) issues and was within the scope of the existing legislative scheme (Office of the Gene Technology Regulator, 2013). Also in 2018, the Australian scientific community reported the outcomes of a horizon scanning process, calling for the already progressive and effective regulatory framework to remain so, by timely responding to technological developments and ensuring regulation that is proportionate to risk (Gray et al., 2018).

### Regional Developments

At the regional level, an early assessment by the European Academies Science Advisory Council (EASAC) in 2010 concluded that the regulatory frameworks that govern safe synthetic biology research and development are already in place or can readily be adapted to cope with the scientific advances foreseen (European Academies Science Advisory Council [EASAC], 2010). Mid-decade, a larger assessment was published by the European Commission in three “opinion” documents prepared by the Scientific Committees on Consumer Safety, on Emerging and Newly Identified Health Risks, and on Health and Environmental Risks. These opinions proposed an operational definition (Scientific Committees, 2014), examined the adequacy of European risk assessment practices for GMOs (according to the applicable regulatory framework) (Scientific Committees, 2015a), and research priorities (Scientific Committees, 2015b). The proposed operational definition coincided with work under the CBD on an operational definition (see section Synthetic Biology Under the CBD below), with that proposed by the Scientific Committees providing the basis for further elaboration by the CBD’s AHTEG on Synthetic Biology in 2015^17^. Notably, on the topic of risk assessment, the Scientific Committees concluded that existing methodologies established for GMOs are adequate, and they made recommendations for research to improve knowledge for the purposes of risk assessment in regard to the particular developments they considered (genetic parts, minimal cells, protocells, xenobiology, DNA synthesis and genome editing, citizen science), and to ensure proportionate regulation with technological advancement (Scientific Committees, 2015a).

More recently in 2018, the European Commission has mandated the European Food Safety Authority (EFSA) to: (i) reflect whether and which newer sectors/advances should be considered among synthetic biology developments in addition to the six identified by the three Scientific Committees; (ii) to identify, where possible, potential risks in terms of impact on humans, animals and the environment for current or near future synthetic biology developments and to identify novel hazards as compared to established GMO techniques; (iii) to determine whether the existing European guidelines for risk assessment are adequate and sufficient for current and near future synthetic biology developments or whether there is a need for updated guidance; and (iv) in case guidance need to be updated, to identify the specific areas where such update is needed. While the publication of a final opinion after public consultation is expected by the end of 2020, the outcome of a literature search conducted by the German Julius Kühn-Institute as part of this work on synthetic biology developments in plants was briefly presented at an EFSA update meeting in June 2019^18^, and it indicates that developments in the agri-food sector are “currently less advanced than in microorganisms” and that many scientists would not recognize plant metabolic engineering as a synthetic biology application.

### International Developments

#### Organisation for Economic Development (OECD)

In recognition of the many potential applications of “synthetic biology” across a range of economic sectors, in 2014 the OECD published a report examining the associated policy issues (Organisation for Economic Cooperation and Development [OECD], 2014). This report highlights that the field benefits from the principles of risk assessment and “decades of regulation and governance” already developed for GMOs, with many experts considering that this is sufficient for synthetic biology as it is not significantly different from GM. It also points out that the potential benefits of synthetic biology may be hindered in some parts of the world due to over-regulation deterring investment in research and development (Organisation for Economic Cooperation and Development [OECD], 2014). An earlier OECD report examined the potential impact of developments in enabling technologies in biological sciences, including the then emerging field of synthetic biology, on industrial biotechnology. The combination of new enabling technologies with fermentation and biochemical engineering was considered to be a driver of economic development, however concerns regarding acceptance of GM were also recognized as a potential barrier to economic development (Organisation for Economic Cooperation and Development [OECD], 2011).

#### International Union for the Conservation of Nature (IUCN)

In the conservation biology field, some practitioners have expressed hope for a convergence between the traditional past-looking conservation mindset and the forward-looking optimism of synthetic biology, with speculation that it could contribute to saving endangered species and even reviving and restoring extinct species (Redford et al., 2013, 2014). Underlying this hope is recognition that new approaches and strategies are needed to address biodiversity loss that continues despite the application of conservation efforts. Applications of synthetic biology that are intended to have direct effects on biodiversity are therefore regarded by some as having great potential for addressing intractable conservation problems, such as the use of gene drives to control invasive species (Piaggio et al., 2017).

The optimism expressed by some is not shared by the all members of the conservation community, with some expressing deep concern (e.g., Civil Society Working Group on Gene Drives^19^). This led to a resolution at the 2016 IUCN World Congress to develop an IUCN policy on biodiversity conservation and synthetic biology^20^, with a Task Force and Technical Subgroup on Synthetic Biology established to support this work. As a contribution toward the beginning of this process, the IUCN commissioned an assessment of the state of science and policy around synthetic biology techniques, including gene drives, as they relate to biodiversity, resulting in a recently published report (International Union for the Conservation of Nature and Natural Resources [IUCN], 2019). The assessment aimed to provide a clear understanding, based on the best available evidence, of synthetic biology issues that are relevant to and may have an impact on the conservation and sustainable use of biological diversity in order to inform future deliberations (International Union for the Conservation of Nature and Natural Resources [IUCN], 2019).

The IUCN report sheds light on tensions in the synthetic biology discussion in that forum that also exist under the CBD (see the following sections): polarized views on the safety versus danger of GMOs, and of their potential beneficial versus adverse effects on biological diversity. The report states that a major concern articulated by groups who are critical of conservation applications of synthetic biology is that they may serve as “Trojan horses” for other “more questionable” applications. In an attempt to address the topic without conflation of many different applications into one for adverse “summary judgment,” the report takes a case-by-case approach and examines eight case studies with a conservation aim, or with a different aim but with impacts on conservation goals. The report also makes a plea for the policy debate to be grounded in evidence, emphasizing that conservation practice “needs to be rigorous and defensible, building on impartial standards that are free from ideology or political bias yet transparent in its advocacy for the natural world” (International Union for the Conservation of Nature and Natural Resources [IUCN], 2019).

The IUCN work on synthetic biology is running in parallel to the synthetic biology program of work under the CBD, and overlapping and cross-cutting programs of work under its Protocols. The IUCN holds a World Conservation Congress every 4 years, with the next one to be held in June 2020 where the draft IUCN synthetic biology policy will be brought to vote (International Union for the Conservation of Nature and Natural Resources [IUCN], 2019). The outcomes of the synthetic biology and gene drive discussions at the World Conservation Congress will likely have an influence on the CBD COP15 that will follow soon after in October 2020. This influence was evident in 2016, following the IUCN resolution calling for the synthetic biology assessment, as this resolution also called for gene drive research for conservation purposes to not be supported until this assessment was done. This was promoted as support for global moratorium on gene drive research in (so far unsuccessful) campaigns^21^ for a COP decision supporting a moratorium on gene drive research (Callaway, 2016).

## Synthetic Biology Under the CBD

The present status of “synthetic biology” in CBD discussions is that it falls within the CBD’s broad definition of “biotechnology” (Article 2; see Figure 2), and “most organisms” developed or currently in development “through techniques of synthetic biology” are considered to be LMOs as defined by the Cartagena Protocol (Article 3; see Figure 2), and that for some organisms this may not be clear, such as “transiently modified organisms” and those developed using certain applications of genome editing (see the 2019 report of the AHTEG on Synthetic Biology^22^).

**FIGURE 4:**
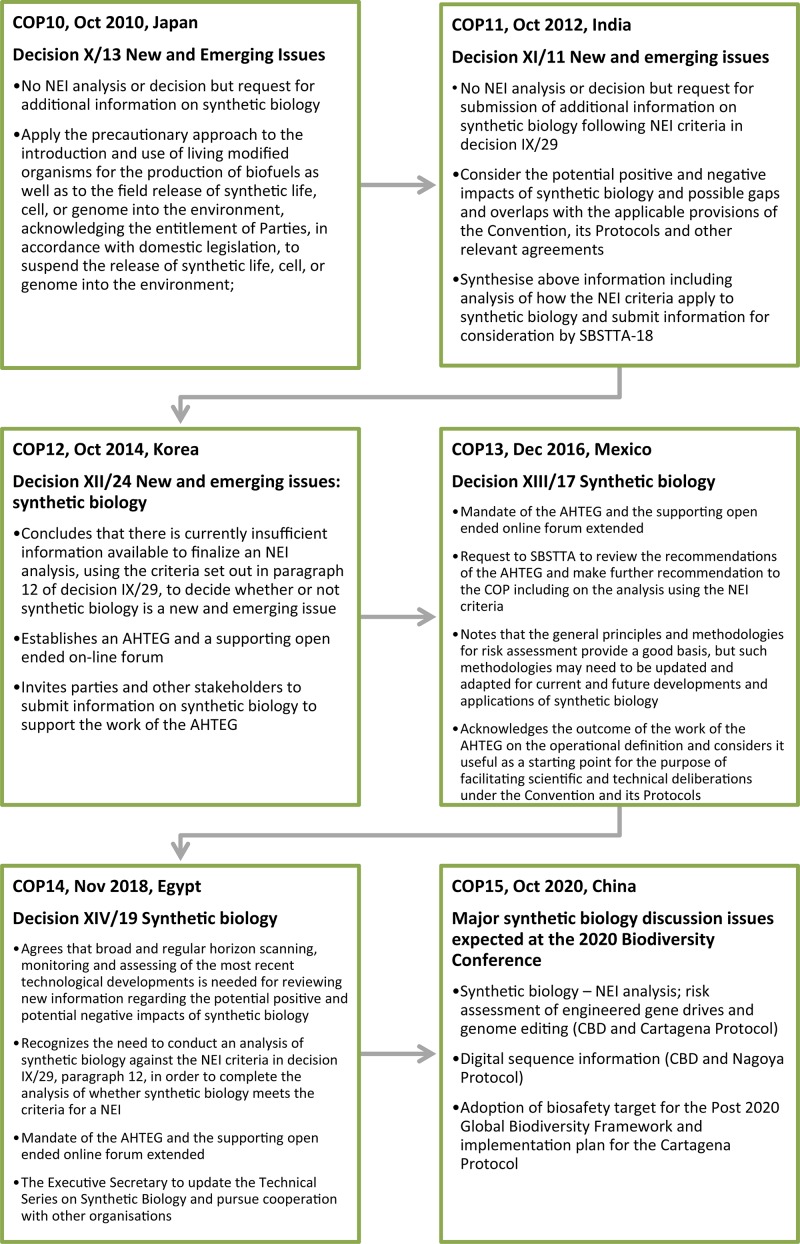
Timeline and highlights of synthetic biology and related NEI COP decisions.

These are the conclusions drawn from an extensive body of work that began in 2010 (see Figure 4) and is ongoing. In 2011 a group of civil society organizations called for urgent consideration of synthetic biology via the CBD’s mechanism for proposing NEIs. These proposals claimed absent or insufficiently comprehensive regulatory oversight, or inadequate regulatory mechanisms for assessing risk, and called for bans on environmental releases and commercial approvals of LMOs developed via synthetic biology until risk and adequacy of regulatory oversight were examined (e.g., EcoNexus^23^; ETC Group^24^; International Civil Society Working Group on Synthetic Biology^25^).

**FIGURE 5:**
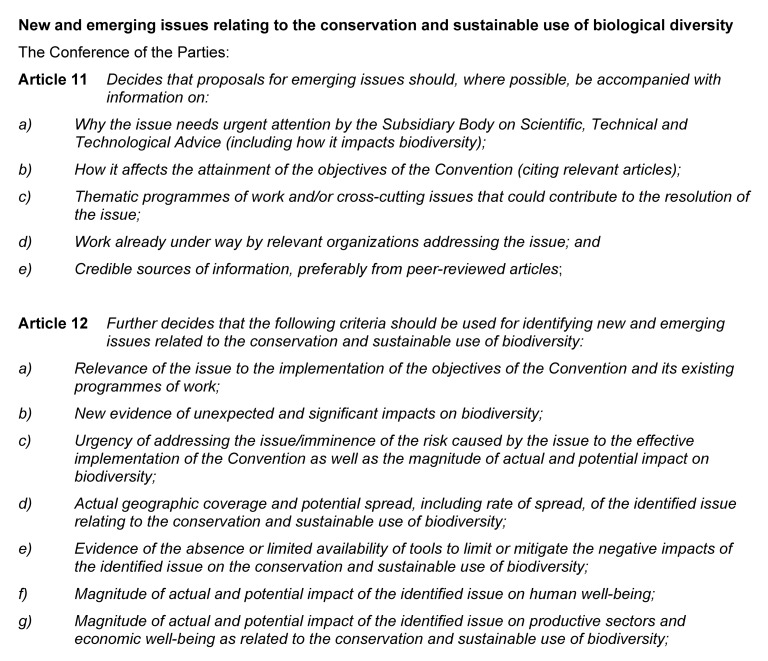
The NEI criteria from COP9 Decision IX/29.

At COP11 that followed these first NEI proposals, and all subsequent COPs, Parties and other governments have been urged to apply a “precautionary approach” with synthetic biology (COP11, Decision XI/11; COP12, Decision XII/24; COP13, Decision XIII/17) or gene drives (COP14, Decision XIV/19), reflecting the preambular language of the CBD (see Figure 2). The CBD synthetic biology program of work began with the COP11 decision in 2012 inviting submissions of information addressing the criteria for identifying a NEI (Decision XI/11). These criteria were established by a prior COP and are set out in Figure 5 (COP9 in 2008; Decision IX/29). The information collected from these submissions was considered at a subsequent meeting of SBSTTA (SBSTTA18) which concluded there was insufficient information to finalize an NEI analysis for a decision on whether or not synthetic biology is a NEI (Recommendation XVIII/7; Decision XII/24). There has not been consideration of synthetic biology against the NEI criteria by a meeting of the SBSTTA since then, however this is expected to be reconsidered at SBSTTA24 in May 2020. If SBSTTA24 makes a recommendation on the topic, it will be followed by deliberation and a decision by the CBD Parties at COP15 in October.

**FIGURE 6:**
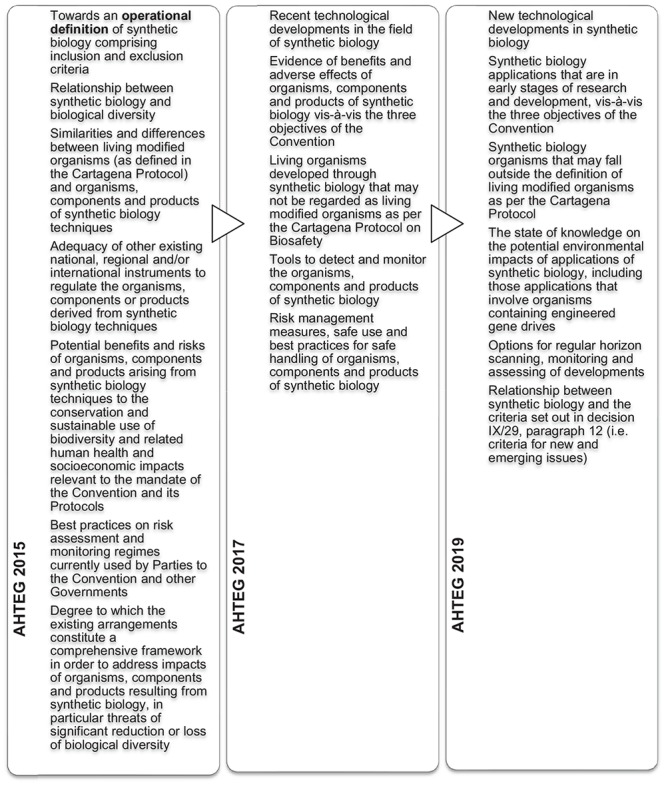
Summary of issues addressed by the AHTEG on Synthetic Biology in 2015, 2017 and 2019.

At COP12 in 2014, the CBD Parties decided, in addition to further submissions of information on synthetic biology, to establish an “open-ended online forum” for a series of discussions on synthetic biology topics, and to establish an AHTEG on Synthetic Biology to deliberate all of the information received and make recommendations for consideration by the next meeting of the SBSTTA (Decision XII/24). This marked a significant expansion of the CBD’s synthetic biology program of work despite there being no recommendation from a SBSTTA meeting or a COP decision that it is in fact a NEI requiring such attention. The program of work is notable for at least two reasons; firstly while the decision highlighted that the Parties “await” a “robust assessment” of synthetic biology against the NEI criteria, it did not attempt to directly address these. Secondly, it mandated the AHTEG to develop an “operational definition” (see Figure 6), the outcome of which was controversial and never formally adopted or endorsed by the CBD Parties at SBSTTA20 (IISD, 2016a) or COP13 (IISD, 2016b). The COP13 decision in 2016 “acknowledges” it as an outcome of the work of the AHTEG that is considered to be a “useful as a starting point for the purpose of facilitating scientific and technical deliberations” under the CBD and its Protocols (Decision XII/17). Despite the dissatisfaction with the definition, it has not been addressed again in subsequent synthetic biology work programs.

The incomplete NEI analysis is another point of contention for some Parties, who have increasingly expressed their dissatisfaction in recent meetings of the SBSTTA where synthetic biology was on the agenda (e.g., SBSTTA20, IISD, 2016a; and SBSTTA22, IISD, 2018). The COP decisions that followed called for further online discussions and submissions of information, and extensions of the AHTEG on Synthetic Biology in programs of work that largely expanded on or duplicated the topics that were deliberated in the previous intersessional periods. However, the terms of reference for the AHTEG on Synthetic Biology in those decisions included the NEI analysis: the COP13 decision in 2016 included an “analysis” by the AHTEG against the NEI criteria (Decision XIII/17), and the COP14 decision in 2018 required the AHTEG to “provide advice” on the relationship between synthetic biology and the NEI criteria (Decision XIV/19).

At the AHTEG on Synthetic Biology meeting in December 2017 (following COP13) the mandated NEI “analysis” was deferred pending clarification from the SBSTTA on how the NEI criteria should be applied. This topic was on the agenda of the subsequent SBSTTA21, but no recommendations were made (Recommendation XXI/7). The CBD Secretariat then prepared a document titled “Analysis against the criteria set out in paragraph 12 of decision IX/29,” whereby text was taken from the AHTEG meeting reports from 2015 and 2017 and allocated to the NEI criteria where it was considered to have relevance. This document was controversial given that the AHTEG’s deliberations and reports did not specifically address these criteria, hence the text used by the Secretariat could have been taken out of context, and not reflect the views of the entire AHTEG on Synthetic Biology^26^. At the AHTEG on Synthetic Biology meeting of June 2019 (following COP14), each of the paragraph 12 criteria were finally deliberated. The criteria were also included in the topics for submissions of information and the online discussions in the broader program of work. The expectation that the SBSTTA24 in May 2020 will revisit this outstanding analysis is based on this collection of information directly addressing the criteria.

Despite the formal NEI process being bypassed to date, synthetic biology has become a fixture on the CBD agenda, particularly since COP12, where there has been extensive debate about the adequacy of existing regulatory oversight for biotechnology and its potential positive and potential negative impacts. A criticism of this debate include its focus on hypothetical applications of “new” technologies rather than actual or realistically foreseeable and technically plausible applications (e.g., see CBD submissions by the Global Industry Coalition^27^). For example, the 2019 meeting of the AHTEG on Synthetic Biology considered “synthetic biology applications that are in the early stages of research and development,” and the meeting report^28^ includes a list compiled from various sources such as research proposals (e.g., environmental applications of engineered bacteria, Gumulya et al., 2018), early stage research reports (e.g., engineering coral, Cleves et al., 2018), and first demonstrations of technology (e.g., gene drive mechanisms in a mammal, Grunwald et al., 2019; and an agricultural pest, Buchman et al., 2018). Another criticism is that demands for expansion of risk assessment requirements disregard the existing experience and accumulated knowledge regarding LMO risk assessment, and the existence of biotech regulatory frameworks and other applicable regulatory mechanisms at international, regional and national levels (CBD submissions by the Global Industry Coalition). The current major issues expected to be debated at the upcoming CBD meetings in 2020 are detailed in the following section.

## Major Synthetic Biology Issues at the 2020 Biodiversity Conference

As referred to above, in October 2020 the governing bodies of the CBD (COP15) and its Protocols (COP-MOP10 for the Cartagena Protocol; COP-MOP4 for the Nagoya Protocol) will meet in concurrent sessions held over a 2-week period in Kunming, China. Synthetic biology is a stand-alone agenda item under the CBD, and it will also be considered within the NEI agenda item. Deliberations on these two agenda items will be based on draft recommendations produced at SBSTTA24 in May 2020.

### New and Emerging Issue Analysis

If the outstanding NEI analysis is addressed at SBSTTA24 as anticipated, and is then the subject of a decision at COP15, this is expected to be one of the most contentious CBD synthetic biology topics at the 2020 Biodiversity Conference. There is a divergence of views amongst Parties as to whether or not the analysis is actually needed to justify continued synthetic biology discussions under the CBD. Some Parties are resistant to completion of the formal analysis and of the view that the topic is clearly relevant to the CBD’s objectives and of sufficient importance to be addressed under the CBD and its Protocols (e.g., see the 2019 NEI proposal by Norway^29^). Conversely, there are Parties of the view that in the absence of this completed analysis, the establishment, continuation and expansion of the CBD synthetic biology program of work cannot be justified, particularly when there are many other obligations parties have, and commitments they have agreed to that are aligned with the CBD’s strategic plan, such as the Aichi Targets, that require extensive resources (e.g., see the 2019 submissions of information by Australia and Brazil^30^). At the 2020 Biodiversity Conference, CBD Parties are expected to adopt an ambitious new strategic plan, currently referred to as the Post-2020 Global Biodiversity Framework, which will also likely require implementation and evaluation of new targets at national levels^31^.

The 2019 submissions of information^32^, online discussions^33^ and report of the AHTEG on Synthetic Biology^34^ also demonstrate that a broad range of views exist on each of the NEI criteria, from these being satisfied by “synthetic biology” as a general concept (e.g., the 2019 NEI proposal by Norway, and the 2019 submissions of information by Finland and Malaysia^35^), or that specific applications such as gene drives (e.g., the 2019 submission of information by Bulgaria) and genome editing (e.g., the 2019 submission of information by Austria) qualify as NEI, to synthetic biology not meeting the NEI criteria (e.g., the 2019 submissions of information by Japan and New Zealand). There are also a range of views as to how the NEI criteria should be applied, e.g., how many of them need to be considered, their relative weighting, and how many of them need to be satisfied before something can be identified as a NEI.

### Gene Drives

The topic of gene drives features prominently in synthetic biology CBD discussions, including any and all possible actual or conceptual applications, e.g., insects, mammals and plants (National Academies of Sciences Engineering and Medicine [NASEM], 2016b; Australian Academy of Science [AAS], 2017; Barrett et al., 2019), and types of drives, and is arguably one of the major drivers of the present-day CBD debate. It is also likely to feature in any deliberation on the NEI issue in the 2020 meetings. While there is no consensus that gene drives are “synthetic biology,” there appears to be a general consensus that organisms containing engineered gene drives are LMOs within the scope of the Cartagena Protocol^36^. This means that organisms containing engineered gene drives are within the scope of LMO regulatory frameworks at the international level, and at regional and national levels of Parties that have implemented the Cartagena Protocol.

The COP14 decision of the CBD Parties in 2018 called upon Parties (and other governments) to “apply a precautionary approach” when considering introducing organisms containing engineered gene drives into the environment, with such decisions to be based on scientifically sound case-by-case risk assessments, and with risk management measures in place to avoid or minimize potential adverse effects (Decision XIV/19). In parallel, Cartagena Protocol Parties made a decision at COP-MOP9 in 2018 under the agenda item of LMO risk assessment and risk management (Articles 15, 16, and Annex III) recognizing that risk assessment guidance for organisms containing engineered gene drives may need to be developed to assist regulators (Decision IX/13). This gave rise to a parallel program of work under the Cartagena Protocol for determining whether or not there is a need to develop such guidance. The work on this topic began with submissions of information^37^, studies commissioned by the Secretariat^38^, and discussions of the open-ended online forum^39^. In March 2020 the AHTEG on Risk Assessment and Risk Management will meet to deliberate all of the information received and make recommendations for consideration by SBSTTA24^40^, which will be the basis of a decision by the Parties at COP-MOP10.

The question of whether or not risk assessment guidance is necessary may seem innocuous, however historically this has been a controversial issue under the Cartagena Protocol, with criticism of the process for the development of guidance materials and the utility of its outcomes (Hokanson, 2019). This controversy led to the establishment of a formal process for the “identification and prioritization of specific issues of risk assessment of living modified organisms that may warrant consideration” (Decision IX/13 Annex I). The current program of work, in effect, is testing the process by applying a defined set of criteria to organisms containing engineered gene drives and to LM fish. The AHTEG will conduct an analysis and make a recommendation regarding whether or not there is a need to develop guidance, as well as recommendations on any adjustments that should be made to the criteria for prioritization of issues for risk assessment.

The submissions of information in 2019 on this topic^41^ indicate that Parties and other governments have not yet received any applications for environmental release of organisms containing engineered gene drives, and hence there is limited direct experience in conducting risk assessment of such organisms. Some regulatory agencies are in the process of reviewing or have already reviewed their procedures for research with gene drive organisms in containment and acknowledge that the general principles and methodology for risk assessment and management, experience from LMO risk assessment, as well as knowledge from fields such as biocontrol agents and invasive alien species, will be relevant to performing risk assessment of organisms containing engineered gene drives. Challenges that are anticipated when performing environmental releases of such organisms are mainly related to the fact that the technology is targeting wild populations and may be irreversible, and thus the step-wise approach to environmental releases, as practiced with other types of LMOs, may require adaptation.

In regard to the NEI analysis for synthetic biology, the primary concerns that emerge specifically for organisms containing engineered gene drives in the 2019 report from the AHTEG on Synthetic Biology include a perceived lack of control and/or mitigation strategies, and traceability and/or detection tools once they are released into the environment, as well as their potential geographical spread and rate of spread. However, the report also hints at the need to consider such concerns in the broader context and taking into account the potential benefits such as human well-being^42^. This consideration is especially relevant to gene drives given that the most advanced application, with field trial releases into the environment envisaged in the near term, is in mosquitoes for the control of malaria^43^.

As noted above, the question of how to proceed with organisms containing engineered gene drives is contentious in other fora such as the IUCN [see section International Union for the Conservation of Nature (IUCN)], with a 2016 resolution calling for what was, in effect, a moratorium on gene drive research for conservation purposes^44^. In early 2020, the European Parliament voted on a resolution (European Parliament, 2020) calling on the EU Commission and the Member States to support a CBD COP15 decision for a global moratorium on releases of organisms containing engineered gene drives into the environment, including in experimental field trials.

### Genome Editing

Another contentious synthetic biology topic that may be addressed by SBSTTA24 and COP15 is whether or not there are new synthetic biology developments that result in living organisms that are not within the scope of the Cartagena Protocol LMO definition (see Figure 2). The 2019 submissions of information, online discussions and the report of the AHTEG on Synthetic Biology indicate that this is a challenging question to address as it is subject to legal and technical interpretations, e.g., the content of the Cartagena Protocol definition of “modern biotechnology” (see Figure 2). It is also evident that it is subject to societal/community values, and how Parties apply the precautionary principle. Views differ most on this topic in regard to organisms developed via certain genome editing applications, as well as “transiently modified organisms,” with relatively less attention paid to non-living “entities” such as protocells.

For living organisms developed using genome editing techniques, the same question has been or is currently being examined at regional and national levels, toward the aim of providing clarity regarding the scope of LMO/GMO regulation. A number of Cartagena Protocol parties and other governments have created exclusions for certain categories of genome editing technologies or products where these could have also been obtained through spontaneous processes or through the use of other (conventional) tools and methods (Dederer and Hamburger, 2019). Those countries have implemented such exclusions based on their implementation of the Cartagena Protocol definition of “modern biotechnology” whereby a “novel combination of genetic material” does not involve DNA changes that could have been obtained spontaneously or with the use of other methods. In these cases, the organism is managed in the same way as other non-LMO organisms. In the CBD synthetic biology discussions, these countries generally disagree that genome editing should be dealt with at the international level (e.g., the 2019 submission of information by Brazil), or that all applications of genome editing could be considered synthetic biology (e.g., the 2019 submissions of information by Australia and New Zealand), or that it requires special consideration within the Cartagena Protocol agenda item of LMO risk assessment and risk management (Articles 15 and 16, Annex III) (see SBSTTA Recommendation XXII/2). Conversely, there are participants in the CBD synthetic biology discussions that view such “non-LMO” organisms as a regulatory gap that needs to be addressed in this forum^45^.

In regard to the NEI analysis for synthetic biology, one of the primary justifications for including genome editing appears to be a perceived lack of availability of detection methods for identification, particularly in regard to organisms developed using genome editing that have few DNA base changes and it may not be possible to distinguish them from other (non-edited) organisms (see the 2019 report of the AHTEG on Synthetic Biology^46^). Cartagena Protocol agenda items for COP-MOP10 that overlap with this discussion include LMO identification (Article 18) in the context of unintentional transboundary movements and emergency measures (Article 17). In 2019, the Online Network of Laboratories for the Detection and Identification of LMOs established under the Cartagena Protocol held online discussions to share their experience on the detection and identification of LMOs developed using genome editing and synthetic biology^47^. In those discussions it was evident that experience and/or technical capabilities are currently lacking in this area, but technologies are continually developing and these could be tested for feasibility.

### Digital Sequence Information (DSI)

Digital sequence information (DSI) is an issue that arose from the CBD synthetic biology discussions, and since 2016 has been a substantial stand-alone agenda item under consideration jointly by the CBD and the Nagoya Protocol. It is expected to be a major topic at the upcoming Biodiversity Convention, where it will also be deliberated in the context of the Post-2020 Global Biodiversity Framework. DSI is a highly polarized issue that is currently under debate in several international fora, including the UN Law of the Sea Convention (UNCLOS), in relation to a new treaty under negotiation that includes marine genetic resources in areas beyond national jurisdiction; the International Treaty on Plant Genetic Resources for Food and Agriculture (ITPGRFA) in relation to plant genetic resources; and it is under evaluation by the World Health Organization in relation to the Pandemic Influenza Preparedness Framework (Manheim, 2016).

Under the CBD, the origin of the DSI issue can be ascertained from the report of the 2015 meeting of the AHTEG on Synthetic Biology^48^, where the “potential adverse effects” of synthetic biology were considered. One such effect listed in that report is the obtaining of benefits from the use of DNA information obtained from a genetic resource without fair and equitable benefit sharing, which is a CBD objective (see Figure 1). The CBD/Nagoya Protocol access and benefit sharing (ABS) regime applies to “users” and “providers” of “genetic resources,” with “genetic resources” generally understood to constitute physical material, such as cell or tissue samples from an organism. A perceived feature of synthetic biology is increasing use and exchange of DNA sequence information without the need for each user of that information to access the source physical resource to which CBD/Nagoya Protocol ABS obligations apply (e.g., subject to prior informed consent and mutually agreed terms; CBD Article 15), resulting in a form of “misappropriation” of that genetic resource and bypassing of the provisions of the Nagoya Protocol. The report from the 2015 meeting of the AHTEG on Synthetic Biology recommended that the Nagoya Protocol COP-MOP “set up mechanisms” for clarifying this issue as it relates to ABS.

Additional commentary in the report of the 2015 meeting of the AHTEG on Synthetic Biology points to a “shift in the understanding of what constitutes a genetic resource,” and this lies at the heart of the continuing CBD/Nagoya Protocol debate. Views on this are highly polarized, with some Parties of the view that the definition of “genetic resources” can only refer to tangible material and not intangible information, whereas other Parties strongly believe that information is within the scope of “genetic resource,” particularly those Parties that view themselves predominantly as “providers” rather than “users” of genetic resources. This user/provider dichotomy is also evident in the DSI debates under the ITPGRFA and in the development of the new treaty under UNCLOS.

In 2016, the CBD (COP13; Decision XIII/16) and Nagoya Protocol (COP-MOP2; Decision II/14) Parties jointly decided to establish a program of work on DSI which included submissions of information, a commissioned study, and an AHTEG on Digital Sequence Information, with the outcomes of that work to be considered by SBSTTA. “DSI” is itself another undefined term, and this first program of work was focused on examining terminology and different types of DSI, and its relationship with the objectives of the CBD and Nagoya Protocol. While the initial discussions on DSI appeared to apply specifically to electronic DNA sequence information, the 2018 report of the AHTEG on Digital Sequence Information contains a broad list of information “relevant to the utilization of genetic resources”^49^. This ranged from genetic and biochemical information that may be obtained from a (physical) genetic resource, to “observational” information associated with it, e.g., ecological relationships, taxonomy, phenotype.

The outcomes of the first DSI program of work were extensively debated at the 2018 meeting of SBSTTA22, as evident by the heavily bracketed text in the resulting recommendation that reflected the lack of consensus amongst the Parties (Recommendation XXII/1). This recommendation was the basis for further contentious debate at COP14/COP-MOP3 later that same year. The eventual decision of COP14 recognizes the divergence of views amongst Parties, and sets out a “science- and policy-based process” aimed at assisting the Parties to work to resolve this (Decision XIV/20). The process includes further submissions of information^50^, four commissioned studies, and extension of the AHTEG on Digital Sequence Information. The topics to be examined in the program of work were aimed at improving “conceptual clarity,” including: terminology and scope, traceability and use of public databases, benefit sharing arrangements for commercial and non-commercial (i.e., research) uses of DSI, and how DSI is considered within existing domestic ABS measures. The COP14 and COP-MOP3 decisions (Decision III/12) refer the outcomes of the work of the AHTEG on Digital Sequence Information to the Open-Ended Working Group on the Post-2020 Global Biodiversity Framework, rather than SBSTTA, who are to submit the outcomes of their deliberations to COP15/COP-MOP4 in October 2020.

At the time of writing, the program of work is in progress, with drafts of the four commissioned studies released in late 2019 for “peer review”^51^, and the AHTEG on Digital Sequence Information scheduled to meet in March 2020. To date there has been limited discussion on DSI in the context of the Post-2020 Global Biodiversity Framework, with the draft text of this released in January 2020 referring to ongoing work in this area^52^.

## Conclusion

In this paper we have provided an overview of the major developments in biotechnology regulation since the first discussions on this topic at the 1975 Asilomar conference on recombinant DNA. While the technologies and the range of organisms developed have evolved since then, accompanied by accumulated experience and expertise in assessing and managing risks, the CBD synthetic biology discussions are, in essence, based on the same concerns about safety and appropriate regulatory oversight that brought about the Asilomar conference. These concerns are at the heart of most of the synthetic biology-related discussions that are anticipated at the 2020 Biodiversity Conference, and these are further conflated with broader political and societal issues. Collectively, these have contributed to the ever-expanding CBD synthetic biology work program, the evidence-based NEI analysis remaining incomplete for almost 10 years, and the relatively new dimension of access and benefit sharing in relation to information.

In the view of the authors, the CBD discussions on synthetic biology can be seen as an exceptionally prolonged version of the Asilomar conference. However, an important distinguishing feature is that the CBD discussions are relatively lacking in participation by its practitioners. This is possibly due to the complex and resource-intensive nature of CBD processes, and the fact that these are Party (or government)-led processes and the scientific community can only “observe” unless they are directly engaged by governments. While there are members of the scientific community that contribute to the CBD discussions, stronger involvement is essential to support evidence-based decision-making and the development and/or adjustment of effective, adaptive and proportionate regulation. The optimism and excitement of the scientific community for providing solutions to global challenges with synthetic biology stands in stark contrast to the CBD debates, which have spent little time on acknowledging the demonstrated or supporting the potential contribution of biotechnology toward the achievement of the biodiversity and sustainability objectives at the heart of the CBD.

## Author Contributions

FK and AA conceived and designed the review. FK drafted the manuscript with support from AA who critically reviewed it and also contributed content.

## Conflict of Interest

The authors are employed by BASF, a global research and development company with a diverse range of chemistry-based business segments, and an agricultural business that includes biotech seed products. They participate in the CBD programs of work mentioned in this manuscript via the Global Industry Coalition and the International Chamber of Commerce. Both authors have participated in the AHTEG on Synthetic Biology.

## Footnotes

1 *Convention on Biological Diversity*, Adopted 5 June 1992, 1760 UNTS 69 (entered into force 29 December 1993).

2 See: https://www.cbd.int/history/

3 See: https://www.cbd.int/information/parties.shtml

4 See: https://www.cbd.int/decisions/

5 See: https://www.cbd.int/sbstta/

6 See: https://www.cbd.int/sbi/

7 Item 4, document CBD/SBSTTA/24/1.

8 See: https://bch.cbd.int/synbio/submissions/

9 See: https://bch.cbd.int/synbio/open-ended/discussion/

10 Document CBD/SYNBIO/AHTEG/2019/1/3.

11 Item 5, document CBD/SBSTTA/24/1.

12 See: https://www.cbd.int/

13 E.g., https://www.economist.com/leaders/2010/05/20/and-man-made-life

14 Document CBD/SYNBIO/AHTEG/2015/1/3.

15 See: https://www3.nuseed.com/us/nufarm-welcomes-worlds-first-regulatory-approval-for-plant-based-long-chain-omega-3/

16 See: https://www.darpa.mil/program/insect-allies

17 Document CBD/SYNBIO/AHTEG/2015/1/3.

18 See: https://www.efsa.europa.eu/sites/default/files/event/4.5_Plant%20SynBio%20ERA_WG.pdf

19 See: http://www.synbiowatch.org/gene-drives-letter/

20 Resolution WCC-2016-Res-086: Development of IUCN Policy on Biodiversity Conservation and Synthetic Biology. Available at: https://portals.iucn.org/library/node/46503

21 E.g., http://www.synbiowatch.org/gene-drives-letter/; http://www.etcgroup.org/sites/www.etcgroup.org/files/files/cbd_cop_13_gene_drive_moratorium_briefing.pdf

22 Document CBD/SYNBIO/AHTEG/2019/1/3.

23 Available at: https://www.cbd.int/doc/emerging-issues/econexus-synthetic-biology-2011-013-en.pdf

24 Available at: https://www.cbd.int/doc/emerging-issues/etcgroup-introduction-synthetic-biology-2011-013-en.pdf

25 Available at: https://www.cbd.int/doc/emerging-issues/Int-Civil-Soc-WG-Synthetic-Biology-2011-013-en.pdf

26 E.g., see the online discussion “Topic 7: Relationship between synthetic biology and the criteria set out in decision IX/29,” held 25-31 March 2019. Available at: https://bch.cbd.int/synbio/open-ended/discussion/

27 Available at: https://bch.cbd.int/database/record.shtml?documentid=114285 (2019), and https://bch.cbd.int/database/record.shtml?documentid=112053 (2017).

28 Document CBD/SYNBIO/AHTEG/2019/1/3.

29 Available at: https://www.cbd.int/doc/emerging-issues/ntf-2019-041-submission-norway-en.pdf

30 Available at: https://bch.cbd.int/synbio/submissions/

31 Zero draft document of 6 January 2020 CBD/WG2020/2/3.

32 See: https://bch.cbd.int/synbio/submissions/

33 See: https://bch.cbd.int/synbio/open-ended/discussion/

34 Document CBD/SYNBIO/AHTEG/2019/1/3.

35 Available at: https://bch.cbd.int/synbio/submissions/.

36 Document CBD/SYNBIO/AHTEG/2019/1/3.

37 March 2019; available at: https://bch.cbd.int/onlineconferences/submissions.shtml

38 Available at: http://bch.cbd.int/onlineconferences/studies.shtml

39 January 2020; aee: http://bch.cbd.int/onlineconferences/forum_ra/discussion.shtml

40 See: https://www.cbd.int/meetings/CP-RARM-AHTEG-2020-01

41 Available at: http://bch.cbd.int/onlineconferences/submissions.shtml

42 Document CBD/SYNBIO/AHTEG/2019/1/3.

43 See: https://genedrivenetwork.org/open-letter/

44 Resolution WCC-2016-Res-086: Development of IUCN Policy on Biodiversity Conservation and Synthetic Biology. Available at: https://portals.iucn.org/library/node/46503

45 E.g., see the online discussion “Topic 5: Consider whether any living organism developed thus far through new developments in synthetic biology fall outside the definition of living modified organisms as per the Cartagena Protocol,” held 18-24 March 2019. Available at: https://bch.cbd.int/synbio/open-ended/discussion/?threadid=9602

46 Document CBD/SYNBIO/AHTEG/2019/1/3.

47 See: https://bch.cbd.int/onlineconferences/portal_detection/2019discussions.shtml.

48 Document CBD/SYNBIO/AHTEG/2015/1/3.

49 Document CBD/DSI/AHTEG/2018/1/4.

50 Available at: https://www.cbd.int/dsi-gr/2019-2020/submissions/

51 Available at: https://www.cbd.int/dsi-gr/2019-2020/studies/

52 Document CBD/WG2020/2/3.
